# Survival improvement with sirolimus plus tacrolimus immunosuppression for treatment of bronchiolitis obliterans syndrome after lung transplantation

**DOI:** 10.1016/j.jhlto.2025.100469

**Published:** 2025-12-19

**Authors:** Marniker Wijesinha, Michael Terrin, Si Pham, Aldo Iacono

**Affiliations:** a Northwell Health, and North Shore University Hospital, Manhasset, NY; b University of Maryland School of Medicine, Department of Medicine, Baltimore, MD

**Keywords:** Lung Transplantation, Immunosuppression, Chronic rejection

## Abstract

**Background:**

Chronic rejection, usually manifesting as bronchiolitis obliterans syndrome (BOS), is the leading cause of death among lung transplant patients. Prior lung transplant studies showed higher overall survival and lower BOS incidence associated with sirolimus (SIR) + tacrolimus (TAC) versus conventional mycophenolate mofetil (MMF) + TAC immunosuppression. However, after BOS occurs, it is unknown how immunosuppressive drugs may be linked to survival.

**Methods:**

This study included U.S. lung transplant recipients in the Lung Allocation Score era (starting May 2005), with a BOS diagnosis documented from 2006 to 2020, in the Scientific Registry of Transplant Recipients dataset. Survival was compared between patients receiving MMF+TAC, SIR+TAC, or SIR+TAC+MMF/azathioprine (SIR+TAC+MMF/AZA) after BOS onset, using multivariable adjusted Cox regression and Inverse Probability of Treatment Weighting (IPTW)-adjusted Kaplan-Meier estimates.

**Results:**

SIR+TAC+MMF/AZA (HR=0.60, p=0.03, n=47) and SIR+TAC (HR=0.67, p=0.04, n=95) were associated with better survival than MMF+TAC (n=1012); each group contained patients from >15 centers. IPTW-adjusted survival probabilities for SIR+TAC+MMF/AZA, SIR+TAC, and MMF+TAC, respectively, were, 1-year: 91%, 84%, 80% and 5-year: 50%, 58%, 42%. Within severely affected BOS patients (BOS Grade 3, or FEV_1_ decrease ≥30%/year, or FEV_1_<25% of predicted at BOS documentation), SIR+TAC+MMF/AZA (HR=0.32, p=0.03) and SIR+TAC (HR=0.50, p=0.05) had larger survival advantages over MMF+TAC; the respective survival probabilities were, 1-year: 91%, 70%, 59%, and 5-year: 41%, 35%, 20%.

**Conclusions:**

Sirolimus + tacrolimus immunosuppression may improve survival in BOS patients, especially severely affected patients with BOS Grade 3, or rapidly declining or low FEV_1_. Adding MMF or azathioprine to this combination may further increase short-term survival.

## Introduction

Lung transplantation extends the lives of many end-stage lung disease patients, but median post-transplant survival remains limited to approximately 6 years. A commonly inevitable long-term complication is chronic rejection that results in the development of chronic lung allograft dysfunction (CLAD), most commonly manifesting as bronchiolitis obliterans syndrome (BOS),^1^, ^2^ which entails progressive and frequently fatal lung function decline. The probability of BOS occurring is ∼50% by 5 years post-transplant, and >75% by 10 years.^3^ After BOS occurs, median survival is ∼3 years; steeper rate of lung function decline and earlier onset after transplantation predict worse post-BOS survival. Lung transplant patients receive lifelong maintenance immunosuppression to attempt to prevent BOS, but it is only partially effective, as BOS remains their leading cause of death. Additionally, many patients die of immunosuppression-related consequences including infections and cancers.

No treatments are currently known to completely halt BOS progression. However, certain adjunctive therapies may reduce disease progression at least temporarily, including azithromycin,^4^ alemtuzumab,^5^ anti-thymocyte globulin (ATG),^6^, ^7^ and inhaled liposomal cyclosporine (L-CsA).^8^ Inhaled L-CsA significantly improved long-term survival in a pilot randomized clinical trial of BOS patients,^8^ while azithromycin^9^ and ATG^10^ have been associated with increased survival in single-center retrospective studies of BOS or CLAD patients. In addition to using adjunctive therapies, common practices when BOS occurs are to increase maintenance immunosuppression dosages, or to add or switch maintenance immunosuppression agents.^11^

Conventional maintenance immunosuppression regimens combine a calcineurin inhibitor [CNI] (tacrolimus, or less commonly, cyclosporine), and an antimetabolite (mycophenolate mofetil [MMF], or less commonly, azathioprine) which serves as a cell cycle inhibitor. An alternative regimen uses a mammalian target of rapamycin (mTOR) inhibitor (sirolimus, or less commonly, everolimus) as the cell cycle inhibitor, instead of an antimetabolite. Since mTOR inhibitors can impair wound healing if administered immediately after transplant,^12^ mTOR inhibitor use is generally delayed at least 3–12 months after lung transplantation. General benefits of mTOR inhibitors include anti-cancer effects,^13^, ^14^, ^15^, ^16^, ^17^ renal-sparing effects in the presence of CNI dose reduction or withdrawal,^18^, ^19^, ^20^ and reduction of cytomegalovirus (CMV) infections^21^; another potential advantage is anti-aging effects^17^ including increased lifespan as demonstrated in mouse studies.^22^

Regarding BOS specifically, multiple mechanisms by which mTOR inhibitors may be beneficial have been demonstrated in pre-clinical studies. In addition to the most well-established mechanisms of mTOR inhibitors which involve blocking the mTOR protein kinase and thereby reducing cytokine-driven T cell proliferation and B cell proliferation through cell cycle disruption,^23^ mTOR inhibitors (both sirolimus and everolimus) have also exhibited synergistic immunosuppressive activity in combination with CNIs.^24^, ^25^, ^26^ Furthermore, sirolimus promoted immune-inhibitory regulatory T and B lymphocytes (Tregs and Bregs) in a mouse model as follows: sirolimus inhibited pro-inflammatory cytokines and induced Breg infiltration, leading to secretion of anti-inflammatory cytokines, which in turn induced an increase in Tregs.^27^ A study in liver transplant patients also found that sirolimus treatment was associated with amplification of Bregs and Tregs that was partly dependent on an increase in anti-inflammatory cytokines (IL-10 and TGF-β),^28^ and a randomized trial in kidney transplant patients also had a sustained increase in Treg population in the sirolimus-treated group.^29^ Additionally, sirolimus has demonstrated anti-fibrotic effects, including the inhibition of fibrocyte migration into tracheal allografts,^30^ mediated by early-stage protection against epithelial loss and late-stage epithelial regeneration,^31^ in a tracheal transplant mouse model, as well as the inhibition of human lung fibroblasts in a cell line study.^32^ Finally, in a rat model of lung transplantation, early treatment with everolimus prevented the development of BOS provided that moderate or severe acute rejection was not already present.^33^

In lung transplant recipients, two cohort studies of prophylactic sirolimus use (sirolimus initiated in the first year post-transplant) with 10-year follow-up,^34^ one of which was based on national U.S. lung transplant data,^35^ showed major improvements in long-term overall survival and BOS-related endpoints using sirolimus instead of MMF, combined with tacrolimus. Randomized controlled trials comparing sirolimus or everolimus versus MMF or azathioprine in lung transplantation, which have been hampered by high discontinuation rates (perhaps partly due to unfamiliarity with mTOR inhibitors^36^) and short follow-up periods (commonly 2–3 years), have mostly not shown statistically significant differences in BOS incidence or mortality.^36^, ^37^ However, the largest of these trials demonstrated significantly less efficacy failures (death, graft loss, or FEV_1_ decline) with everolimus than azathioprine,^38^ and another trial found that among patients who remained on their assigned treatment without discontinuation, everolimus yielded significantly lower BOS incidence than MMF.^36^ Likewise, in a trial comparing everolimus + reduced dose CNI + antimetabolite vs. full dose CNI + antimetabolite, among patients retained on their assigned regimen, there was a nearly significant trend towards higher CLAD-free survival in the everolimus-treated group.^39^ Additionally, a single-center retrospective study found better lung function in patients initiating sirolimus within 6 months post-transplant compared to patients initiating it later.^40^ Finally, a small single-center study of very early sirolimus initiation (∼1 month post-transplant in patients with healed bronchial anastomoses) reported very low long-term BOS incidence.^41^

Even in patients who already developed BOS, several small single-center studies suggest potential benefits of mTOR inhibitors. The largest such study, consisting of 57 CLAD patients (54 with BOS) who were switched to an everolimus + tacrolimus regimen with or without MMF, reported that the overall FEV_1_ decline in the study population was significantly ameliorated over 1 year following everolimus initiation.^42^ In another study that included 16 BOS patients switched to a sirolimus + tacrolimus regimen, BOS grade improved in half the patients and remained stable in the other half over 1 year.^43^ A study of 11 BOS patients in whom sirolimus was initiated alongside ongoing tacrolimus or cyclosporine reported that 8 of them (73%) experienced stabilization or improvement of lung function over 6–12 months.^44^ Another study which switched BOS patients to sirolimus + tacrolimus did not find a significant change in FEV_1_ slopes over ∼6 months, although patients whose FEV_1_ was declining rapidly tended to experience FEV_1_ stabilization following sirolimus initiation.^45^ Finally, in a study where patients were switched from calcineurin inhibitors to sirolimus alongside ongoing MMF, although FEV_1_ continued deteriorating overall, FEV_1_ decline slowed in about half the patients following sirolimus initiation; the patients transitioned to sirolimus earlier after BOS onset tended to have more favorable outcomes.^46^ In general, the results of these studies suggest that mTOR inhibitors may partly attenuate BOS progression, especially if initiated soon after BOS occurrence, and may offer greater benefits in patients experiencing more rapid BOS progression. However, these studies lacked comparison groups, so it remains possible that lung function would have stabilized during the short observation periods even without mTOR inhibitor treatment. Importantly, these studies were not equipped to assess survival trends associated with mTOR inhibitor administration due to short follow-up periods and the absence of control groups.

Because of the promising results associated with post-BOS use of mTOR inhibitors in these small studies, as well as the positive findings associated with prophylatic mTOR inhibitor use on BOS or CLAD related outcomes in some clinical trials or long-term cohort studies, we hypothesized that using mTOR inhibitors in combination with tacrolimus may improve survival among BOS patients, and that the advantages of mTOR inhibitors might be largest among severely affected BOS patients, as at least one study suggested.^45^ In the national U.S. lung transplant dataset used for the current study, documented post-BOS use of everolimus was very uncommon, precluding reliable analysis; therefore, sirolimus was the only mTOR inhibitor that could be examined in the current study. In addition, beyond the potential benefits of a sirolimus + tacrolimus regimen in BOS, we also hypothesized that an immunosuppressive regimen utilizing all 3 drugs from different classes (sirolimus, tacrolimus, and MMF) might especially benefit BOS patients due to possible advantages of more comprehensive and intensified immunosuppression. Particularly since sirolimus has exhibited synergistic immunosuppressive effects with both tacrolimus^24^, ^25^ and MMF,^47^ it is plausible that utilizing sirolimus in combination with both of these drugs may deliver optimal immunosuppressive efficacy for treating BOS. However, our previous study on prophylactic sirolimus use found that only sirolimus + tacrolimus, but not sirolimus + tacrolimus + MMF, was associated with a survival improvement over MMF + tacrolimus,^35^ suggesting that potential additional toxicities of combining all 3 drugs may be a concern. The primary aim of the current study was to compare post-BOS survival between sirolimus, MMF, or the two drugs together, in combination with tacrolimus. The survival comparisons were performed both among BOS patients overall, and within a subgroup comprised of severely affected BOS patients.

## Methods

### Study design and population

This retrospective cohort study included U.S. lung transplant recipients in the Lung Allocation Score (LAS) era (starting May 2005), with a diagnosis of BOS documented between 2006 and 2020, in the Scientific Registry of Transplant Recipients (SRTR) dataset. The SRTR is the national U.S. organ transplant registry,^48^ and its data are collected by the Organ Procurement and Transplantation Network (OPTN), under contract with the U.S. Department of Health and Human Services (HHS). SRTR data enable assessment of overall outcomes according to patient, donor, and transplant characteristics, including transplant center performance, and treatments and clinical care administered, etc. The data, predominantly reported by transplant centers and subject to a quality validation process, are updated monthly for each transplant patient, as are data from the Social Security Administration Death Master File (SSADMF), for accurate tracking of patient deaths.

**Figure 1:**
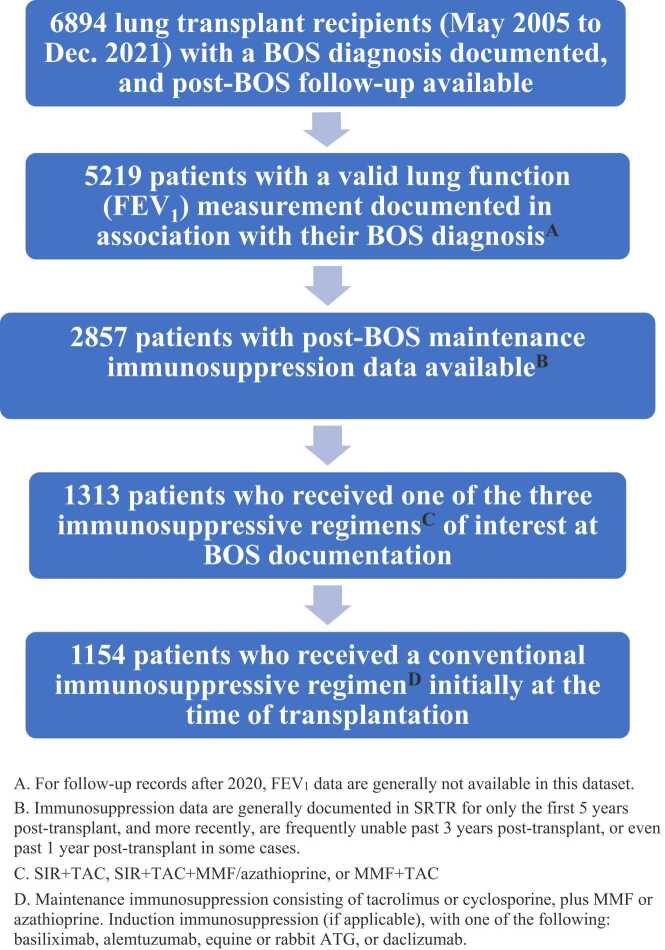
Flow Diagram of Included Patients.

A flow diagram summarizing the inclusion and exclusion criteria for patients in this study is shown in Figure 1. Patients with no post-BOS follow-up (including patients whose BOS was only documented at their death record in the dataset), and patients without a valid FEV_1_ measurement associated with their BOS diagnosis (generally not available after 2020 in this dataset), were excluded. All patients in this study initially received a conventional maintenance immunosuppressive regimen consisting of tacrolimus or cyclosporine, plus mycophenolate mofetil (MMF) or azathioprine, almost universally with corticosteroids. Induction therapy, if administered, utilized one of the following agents: basiliximab, daclizumab, equine or rabbit ATG, or alemtuzumab. A patient’s post-BOS immunosuppression regimen was defined as the maintenance immunosuppression regimen at their initial record following BOS documentation in the dataset. Because immunosuppression data are generally recorded only at yearly intervals in the dataset, some patients who died very soon after BOS diagnosis (before their next yearly follow-up record) had to be excluded due to unavailability of post-BOS immunosuppression data. Furthermore, because immunosuppression data were routinely collected only up to 5 years post-transplant in the dataset, this study was predominantly limited to patients who developed BOS within 5 years post-transplant. For all analyses, patients were retained in their original group based on their initial post-BOS immunosuppression regimen, regardless of any eventual regimen switches.

Post-BOS maintenance immunosuppression regimens compared in this study were: sirolimus + tacrolimus (SIR+TAC), sirolimus + tacrolimus + MMF/azathioprine (SIR+TAC+MMF/AZA), and MMF + tacrolimus (MMF+TAC). The SIR+TAC+MMF/AZA group included patients who received azathioprine instead of MMF, as azathioprine was used as the antimetabolite in some patients receiving a sirolimus + tacrolimus + antimetabolite regimen, and two prior nationwide studies of U.S. lung transplant recipients indicated that survival was as good with azathioprine + tacrolimus compared to MMF + tacrolimus.^35^, ^49^ As an aside, we also assessed survival associated with post-BOS adjunctive use of certain anti-rejection therapies recorded in the dataset, including: equine or rabbit anti-thymocyte globulin (ATG), rituximab, and corticosteroids (besides the maintenance corticosteroids used in nearly all patients). Survival after BOS documentation was the main outcome of interest for this study; the cut-off date for availability of complete follow-up data on patient survival status was June 2023.

### Statistical analyses

All analyses were performed using SAS 9.4 or R 4.4.0. Between the three groups, continuous variables were compared using Wilcoxon rank sum tests (accommodating non-normal distributions) and categorical variables were compared using chi-square tests. In adjusted survival analyses utilizing Cox regression, covariates included age, gender, race, BMI, education, cigarette smoking history, lung disease type at transplant, single or double lung transplant, prior lung transplant, donor age, donor gender, pre-BOS receipt of sirolimus, switch of immunosuppressive regimen at BOS documentation (if available), receipt of adjunctive anti-rejection therapies at BOS documentation (corticosteroids, equine or rabbit ATG, rituximab), BOS grade (if available), FEV_1_ at BOS documentation, FEV_1_ change from maximum preceding BOS documentation (if available), history of acute rejection preceding BOS, prior hospitalization for rejection (if available), hospitalization for infection within 1 year preceding BOS documentation, renal dysfunction within 1 year preceding BOS documentation, post-transplant pre-BOS cancer diagnosis, functional status at BOS documentation, oxygen use at BOS documentation (if available), calendar year of BOS documentation, years since transplantation, and transplant center (random effect).

To enable adjusted comparisons of absolute survival metrics (e.g. median survival and interquartile range, survival probabilities at time points, etc.) between groups while minimizing confounding, we generated adjusted Kaplan-Meier survival curves using Inverse Probability of Treatment Weighting (IPTW).^50^ We first calculated a propensity score (PS) for each patient representing the probability of being in a particular treatment group based on available covariates, using a generalized linear mixed model that included a random effect for transplant center and fixed effects for other major variables. For IPTW-adjustment of Kaplan-Meier survival estimates, each patient was weighted according to the inverse of their PS for the group they are actually in. The IPTW approach has the advantage of including all patients (represented to varying degrees), unlike traditional PS matching, which forces exclusion of patients who cannot be matched to patients in another group.

In addition to overall survival as the main endpoint, we compared the incidence of deaths from common causes (chronic rejection or respiratory failure, infection, and cancer) between groups, noting that some deceased patients were missing cause of death data. We also compared post-BOS FEV_1_ changes between groups while accounting for truncation of FEV_1_ data by death, using two methods. The first method compared the treatment groups based on frequency distributions of ordinal outcome status: [1) alive- with FEV_1_ stability (within ±20%) or increase of >20%; 2) alive- with >20% FEV_1_ decrease, or 3) dead], via the Cochran-Mantel-Haenszel statistic. The second method involved a worst-rank score analysis^51^ based on a Wilcoxon rank sum test, with surviving patients ranked according to FEV_1_ change, and dead patients assigned worse ranks than all surviving patients, with the worst ranks being assigned to the earliest deaths. Since FEV_1_ data are routinely collected in the dataset only up to 5 years post-transplant, and this study is already predominantly restricted to patients who developed BOS within 5 years post-transplant due to data limitations described previously, we analyzed FEV_1_ data at 1 year post-BOS (rather than later on) to enable maximum inclusion of patients.

Finally, focusing on severely affected BOS patients with characteristics linked to especially high mortality, such as: BOS grade 3, or FEV_1_ decline of ≥30% per year preceding BOS documentation (examining FEV_1_ decline was important since some patients were missing BOS grade data), or FEV_1_<25% of predicted, we compared survival according to immunosuppressive regimen within this severely affected patient subgroup. Severely affected BOS patients are of special interest because they have the greatest need for improved treatments due to their considerably higher mortality than BOS Grade 1 patients or BOS patients with higher FEV_1_. Additionally, some evidence suggests that severely affected BOS patients may particularly benefit from sirolimus.^45^

## Results

### Comparison of patient characteristics between groups

Table 1 compares major patient characteristics between the three post-BOS immunosuppression groups: SIR+TAC (n=95), SIR+TAC+MMF/AZA (n=47), and MMF+TAC (n=1012). Overall, the two sirolimus-receiving groups had somewhat sicker patients than the MMF+TAC group, as evident from higher BOS grades (p=0.002) and lower FEV_1_ at baseline (p=0.03). The SIR+TAC group contained the highest proportion of BOS Grade 3 patients (42%), compared to 29% in SIR+TAC+MMF/AZA and 24% in MMF+TAC. Meanwhile, the MMF+TAC group contained the highest proportion of BOS Grade 1 patients (56%), compared to 42% in SIR+TAC and 24% in SIR+TAC+MMF/AZA. The SIR+TAC+MMF/AZA and SIR+TAC groups had lower FEV_1_ % of predicted overall (median [IQR]: 43 [36−54] and 47 [33−63], respectively), compared to 50 [36−66] for MMF+TAC. Additionally, the frequency of adjunctive (non-maintenance) corticosteroid use was higher in the SIR+TAC+MMF/AZA (32%) and SIR+TAC (23%) groups than the MMF+TAC group (15%), which is also potentially indicative of sicker patients being more prevalent in the sirolimus-receiving groups. Post-transplant cancer diagnoses at baseline (prior to BOS documentation) were more prevalent among patients in the SIR+TAC group (13%) and the SIR+TAC+MMF/AZA (17%) group, compared to the MMF+TAC group (6%), p=0.01. The percentage of patients diagnosed with BOS in the more recent era (i.e. 2013–2020 rather than 2006–2012) was lower in the SIR+TAC group (37%) and the SIR+TAC+MMF/AZA group (43%) than in the MMF+TAC group (49%), but the differences were short of statistical significance (p=0.07). Among patients with immunosuppression data available for the follow-up record most recently preceding BOS documentation, 31% in the SIR+TAC group were already receiving SIR+TAC prior to BOS documentation, and 17% in the SIR+TAC+MMF/AZA group were already receiving SIR+TAC+MMF/AZA prior to BOS documentation, while only ∼1% of patients in the MMF+TAC group were receiving either SIR+TAC or SIR+TAC+MMF/AZA prior to BOS documentation.

**Table 1:** Baseline Patient Characteristics (at BOS Documentation)

		SIR+TAC+MMF/AZA	SIR+TAC	MMF+TAC	p-value
		47 Patients 19 Centers	95 Patients 35 Centers	1012 Patients 69 Centers	
Age	Median (IQR), Years	58 (42-63)	57 (43-66)	59 (44-65)	0.78
Gender					0.48
	Female	23 (49%)	38 (40%)	405 (40%)	
	Male	24 (51%)	57 (60%)	606 (60%)	
Transplant Type					0.13
	Single	21 (45%)	39 (41%)	342 (34%)	
	Double	26 (55%)	34 (59%)	669 (66%)	
Previous Lung Transplant					0.50
	Yes	1 (2%)	6 (6%)	44 (4%)	
	No	46 (98%)	89 (94%)	967 (96%)	
Lung Allocation Score (LAS)	Median (IQR)	38 (34-41)	38 (34-44)	39 (35-49)	0.09
BOS Grade*					0.002
	1	4 (24%)	21 (42%)	375 (56%)	
	2	8 (47%)	8 (16%)	133 (20%)	
	3	5 (29%)	21 (42%)	163 (24%)	
FEV_1_ % of Predicted	Median (IQR)	43 (36-54)	47 (33-63)	50 (36-66)	0.03
Percent Change in FEV_1_ % of Predicted Per Year, pre-BOS*	Median (IQR)	-24 (−35 – −11)	-19 (−27 – −9)	-18 (−33 – −8)	0.61
Corticosteroids (non-maintenance) as adjunctive treatment		15 (32%)	22 (23%)	155 (15%)	0.002
Acute Rejection Preceding BOS		28 (60%)	62 (65%)	514 (51%)	0.07
Hospitalization for Rejection Preceding BOS Diagnosis		18 (38%)	34 (37%)	362 (36%)	0.50
Renal Dysfunction in Year Preceding BOS Diagnosis		5 (11%)	22 (23%)	171 (17%)	0.35
Post-Transplant Cancer Diagnosis Preceding BOS		8 (17%)	12 (13%)	64 (6%)	0.01
Era of BOS Diagnosis					0.07
	2006-2012	27 (57%)	60 (63%)	514 (51%)	
	2013-2020	20 (43%)	35 (37%)	498 (49%)	
Years Since Transplant	Median (IQR)	2 (1-3)	2 (2-3)	2 (1-3)	0.004

* Based on patients with available data. BOS grade: 36% missing data; Percent Change/Year in FEV_1_ % of Predicted, pre-BOS: 6% missing data and 37% unavailable due to absence of prior FEV_1_ value in dataset

### Comparison of survival by post-BOS maintenance immunosuppression

**Figure 2:**
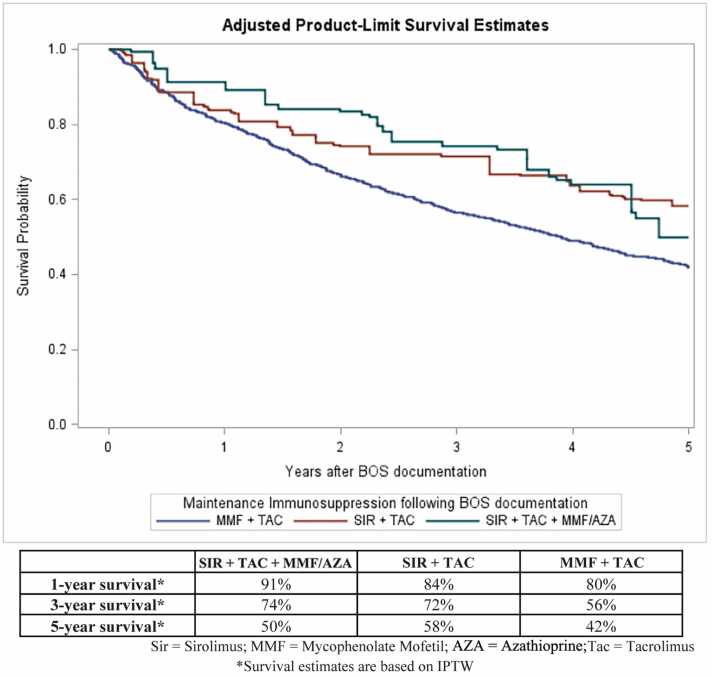
IPTW-adjusted Kaplan-Meier Estimates of Survival after BOS documentation, by Immunosuppressive Regimen.

In adjusted Cox regression analyses, the SIR+TAC+MMF/AZA and SIR+TAC groups had better post-BOS survival than the MMF+TAC group. These results are shown in Table 2. Compared to MMF+TAC, the adjusted Hazard Ratios (HR) were SIR+TAC+MMF/AZA: 0.60 (95% CI: 0.38–0.95), p=0.03; and SIR+TAC: 0.67 (95% CI: 0.45–0.98), p=0.04. IPTW-adjusted Kaplan-Meier survival curves for the three groups are shown in Figure 2. The IPTW-adjusted 1-year survival probabilities for SIR+TAC+MMF/AZA, SIR+TAC, and MMF+TAC were 91%, 84%, and 80%, respectively; the corresponding 5-year survival probabilities were 50%, 58%, and 42%. In the SIR+TAC+MMF/AZA group, 10/47 (21%) received azathioprine instead of MMF; survival was similar with azathioprine (HR=0.93 [95% CI: 0.31–2.83], p=0.90) versus MMF as the third agent.

**Table 2:** Cox Regression Analyses Comparing Mortality after BOS documentation, according to Immunosuppressive Regimen

	SIR + TAC + MMF/AZA	SIR + TAC	MMF + TAC
Adjusted HR	0.60	0.67	1.00
95% CI for HR	(0.38–0.95)	(0.45–0.98)	
p-value	p = 0.03	p = 0.04	Reference
# of Patients	47	95	1012
# of Centers	19	35	69

### Assessment of survival effects of adjunctive (non-maintenance) anti-rejection therapies

Hazard Ratios assessing the survival impact of other adjunctive anti-rejection immunosuppressive therapies: equine or rabbit anti-thymocyte globulin (ATG), rituximab, and (non-maintenance) corticosteroids, are shown in Table 3. None of these therapies had a statistically significant association with survival. However, there was a trend towards improved survival with adjunctive Equine ATG: HR=0.50 (0.22–1.13), p=0.10.

**Table 3:** Survival Associated with Adjunctive (non-maintenance) Anti-Rejection Therapies for Treating BOS

Anti-Rejection Therapy	Adjusted HR (95% CI) for use vs. non-use
Equine ATG, n=19	0.50 (0.22-1.13), p=0.10
Rabbit ATG, n=77	1.10 (0.79-1.54), p=0.57
Rituximab, n=26	1.05 (0.58-1.89), p=0.87
Corticosteroids (non-maintenance), n=192	1.06 (0.83-1.41), p=0.56

### Comparison of risks of major causes of death, by maintenance immunosuppressive regimen

53% of deaths were due to chronic rejection or respiratory failure. There was a significantly lower risk of death due to chronic rejection or respiratory failure among patients receiving SIR+TAC+MMF/AZA: HR=0.35 (95% CI: 0.16–0.75), p<0.01, or SIR+TAC: HR=0.46 (95% CI: 0.24–0.85), p=0.01, compared to MMF+TAC. Deaths due to infection or cancer each occurred in <15% of patients in all groups. There were no significant differences between the groups in the risks of death due to infection or cancer.

### Comparison of FEV_1_ change in year 1 between groups, accounting for truncation by death

**Figure 3:**
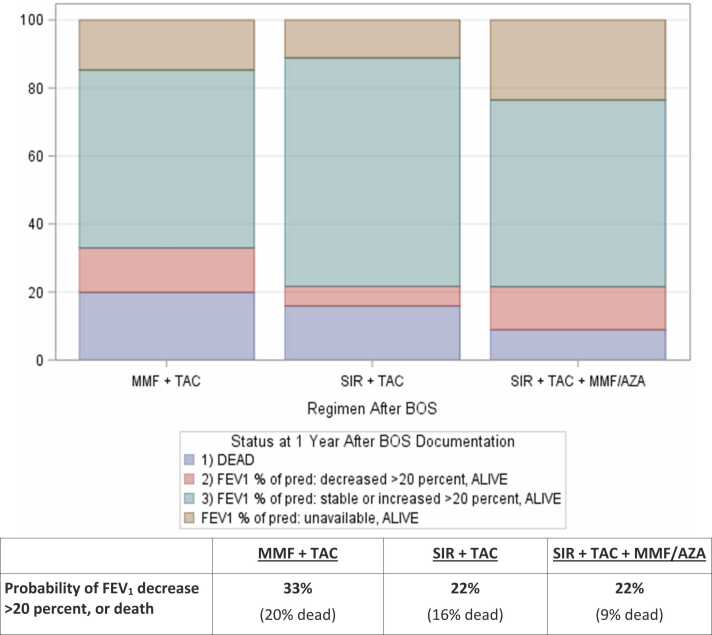
Outcome Status at 1 Year Post-BOS, by Immunosuppressive Regimen.

At 1 year after BOS documentation, the percentages of patients who had an FEV_1_ decline of >20% (relative to FEV_1_ at BOS documentation) or died were: 22% for SIR+TAC+MMF/AZA, 22% for SIR+TAC, and 33% for MMF+TAC, p<0.01. The distribution of ordinal outcome status at 1 year by treatment group [1) alive- with FEV_1_ stability (within ±20%) or increase of >20%; 2) alive- with >20% FEV_1_ decrease, or 3) dead], also including a category for patients alive but missing post-BOS data, is shown in Figure 3. A worst-rank score analysis also indicated that overall, patients in the SIR+TAC+MMF/AZA and SIR+TAC groups had higher probabilities of survival with FEV_1_ stability or improvement at 1 year, compared to the MMF+TAC group (p<0.001).

### Comparison of survival by post-BOS maintenance immunosuppression, in severely affected patients

**Figure 4:**
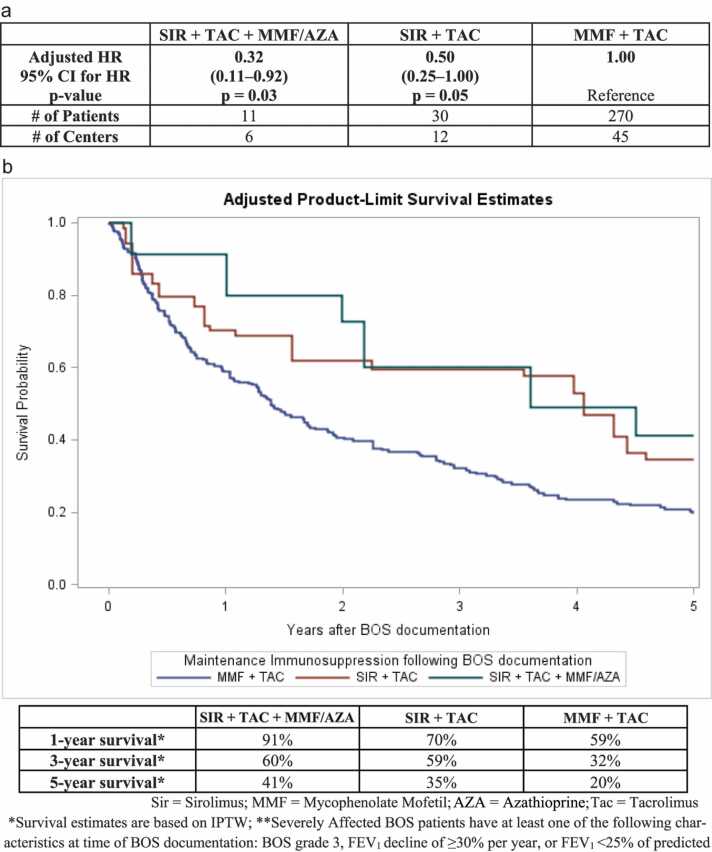
a: Survival Analyses Comparing Immunosuppressive Regimens in Severely Affected** BOS patients. b: IPTW-adjusted Kaplan-Meier Survival Estimates among Severely Affected** BOS patients, by Immunosuppressive Regimen.

Within patients who were severely affected at the time of BOS documentation (BOS grade 3, or FEV_1_ decline of ≥30% per year, or FEV_1_<25% of predicted), the median survival was 1.4 [IQR: 0.4–3.8] years with MMF+TAC immunosuppression. Among severely affected BOS patients, SIR+TAC+MMF/AZA [HR=0.32 (95% CI: 0.11–0.92), p=0.03] and SIR+TAC [HR=0.50 (95% CI: 0.25–1.00), p=0.05] were associated with better survival than MMF+TAC in adjusted analyses; the magnitude of survival improvement associated with the sirolimus-containing regimens was larger in these patients than in BOS patients overall. These results are shown in Figure 4a. The corresponding IPTW-adjusted survival curves are shown in Figure 4b. The IPTW-adjusted 1-year survival probabilities for SIR+TAC+MMF/AZA, SIR+TAC, and MMF+TAC were 91%, 70%, and 59%, respectively; the 5-year survival probabilities were 41%, 35%, 20%.

## Discussion

As the leading cause of death after lung transplantation, Bronchiolitis Obliterans Syndrome (BOS) lacks effective treatments, and this is a major contributor to the poor long-term survival of lung transplant recipients. Using national U.S. lung transplant data, this study compared survival among lung transplant patients with BOS according to the maintenance immunosuppressive regimen received following BOS documentation. Three different tacrolimus (TAC) based regimens were compared, based on their inclusion of sirolimus (SIR) and/or mycophenolate mofetil (MMF) as additional agents: SIR+TAC, MMF+TAC, and SIR+TAC+MMF/AZA (for a SIR+TAC+MMF regimen, azathioprine could substitute MMF). To our knowledge, no prior study has compared long-term survival between BOS patients receiving sirolimus-containing regimens versus the conventional MMF+TAC regimen.

This study found that both SIR+TAC and SIR+TAC+MMF/AZA were associated with significantly better survival than MMF+TAC in adjusted analyses. In the first 2–3 years after BOS documentation, SIR+TAC+MMF/AZA exhibited a survival advantage over SIR+TAC, which largely disappeared later on, while both sirolimus-containing regimens still maintained a survival advantage over MMF+TAC at 5 years after BOS documentation. For both sirolimus-containing regimens, the magnitude of survival advantage over MMF+TAC was larger within the severely affected BOS patient subgroup than among BOS patients overall, consistent with prior research suggesting that sirolimus may especially benefit patients with more severe FEV_1_ decline.^45^ The predominantly early advantage of SIR+TAC+MMF/AZA over SIR+TAC was also more profound within the severely affected patient subgroup. Additionally, the similarity of outcomes with use of azathioprine instead of MMF, as the third agent in the SIR+TAC+MMF/AZA group, indicates that azathioprine may be considered a viable alternative to MMF in this context, potentially offering better tolerability^52^ in certain patients particularly given the possibility of increased toxicity when combining three potent immunosuppressive drugs.

Analyzing common causes of death among the three groups, we found that SIR+TAC+MMF/AZA and SIR+TAC were both associated with large reductions in deaths from chronic rejection or respiratory failure (which comprised over half of the deaths in this population), compared to MMF+TAC. Deaths from infection and cancer were relatively infrequent and did not differ significantly between the three groups. The hypothesis that sirolimus may improve survival by mitigating the pathogenesis of chronic rejection also appears to be supported by our findings that patients receiving sirolimus-containing regimens were less likely to experience a ≥20% decrease in lung function (FEV_1_) at 1 year after BOS documentation (accounting for truncation of FEV_1_ data due to death), compared to those receiving MMF+TAC. It is also noteworthy that this study (in BOS patients) replicates the finding of a survival advantage associated with sirolimus in lung transplantation, which we observed in our previous study of a distinct population (prophylactically-treated patients).^35^ Furthermore, this previous finding was largely mediated by a reduction in BOS-related deaths, consistent with the current findings.

Since the improved survival using sirolimus-containing regimens was predominantly attributable to reduced rejection-related deaths, this suggests that adequate sirolimus dosages are likely to be critical to obtain this potential benefit, although risks of overimmunosuppression are also important to consider. While we lack adequate evidence to make recommendations on optimal drug dosages or trough levels since these variables are not available in the dataset, it is worth noting that the overall results of studies of prophylactic sirolimus use suggest that moderately high sirolimus trough levels (approximately 8–10 ng/ml) may be linked to superior overall survival and BOS prevention relative to lower levels. A single-center study of long-term prophylactic SIR+TAC use, which maintained SIR and TAC levels around 9 and 8 ng/ml respectively at year 2 with progressively reduced prednisone dosages, reported 100% avoidance of BOS until 5 years post-transplant, and 67% survival at 10 years.^34^ Similarly, a long-term small cohort study which targeted fairly high sirolimus trough levels (8–12 ng/ml), in conjunction with progressively reduced cyclosporine levels and low prednisone dosages, reported very low long-term BOS incidence and favorable survival.^41^ In contrast, a randomized open-label trial which maintained sirolimus and tacrolimus levels each at 6–7 ng/ml did not find a significant benefit of sirolimus over azathioprine up to 3 years, although the azathioprine group had higher tacrolimus trough levels,^37^ which likely put the sirolimus group at a disadvantage particularly since its sirolimus levels were also lower than in the aforementioned more successful studies. Of note, sirolimus in inhaled form, currently planned for clinical trials to treat lung diseases including BOS,^53^ may obviate the need for high systemic sirolimus dosages and thereby limit toxicity, while potentially enabling greater effectiveness.

Among the adjunctive anti-rejection treatments we examined that were available in the dataset (Equine or Rabbit ATG, rituximab, and non-maintenance corticosteroids), none were significantly associated with survival among BOS patients. There was a trend towards improved survival in patients who received Equine ATG post-BOS, with a highly clinically meaningful HR of 0.5 (corresponding to a 50% mortality reduction) that was not statistically significant owing to the small number of patients. This finding appears to be consistent with two single-center retrospective studies of ATG in CLAD patients.^10^ Conversely, the other adjunctive therapies examined did not exhibit any favorable associations with survival. Besides the potential explanations that these adjunctive treatments lack significant effectiveness, or that their risks negate their benefits, another possibility is that they were used with lower maintenance immunosuppression doses to limit toxicity, and these lower maintenance immunosuppression doses may have offset any potential benefits of the adjunctive treatments. Information on use of extracorporeal photopheresis was not adequately available in the dataset.

This study has multiple limitations. As a retrospective cohort study it is susceptible to confounding, and despite the comprehensive adjustments for many important covariates, it is not possible to completely rule out unmeasured confounding as an alternative explanation for the findings. Nevertheless, it should be noted that the measured confounding predominantly disfavored the sirolimus-treated groups, since at baseline (time of BOS documentation), patients in these groups had worse BOS grades and lower FEV_1_ overall, as well as a higher frequency of prior post-transplant cancer diagnoses. The phenomenon that patients who received sirolimus were sicker overall matches a priori expectations that sirolimus tends to be most commonly used as a “rescue therapy” rather than as part of routine immunosuppression in lung transplantation. Furthermore, the presence of measured confounding mostly disfavoring the sirolimus-treated groups makes it more likely that unmeasured confounding may also generally disfavor the sirolimus-treated groups, and less likely that unmeasured confounding could be completely responsible for the observed survival advantages of sirolimus, though the latter is still a remote possibility.

Another limitation, arising from the fact that sirolimus is not commonly utilized and tends to be typically initiated as a “rescue therapy” following the occurrence of serious conditions, such as high-grade BOS or cancer as apparent in this study, is that only relatively small numbers of patients receiving sirolimus are available. Nevertheless, although use of the SIR+TAC+MMF/AZA regimen was rather uncommon, there were nearly 50 patients from almost 20 different transplant centers in this group, which demonstrates the robustness of the favorable survival using this regimen. Meanwhile, the SIR+TAC group, which also exhibited significantly better survival than MMF+TAC, had nearly 100 patients from 35 different centers. Furthermore, the inevitable exclusion of many patients due to limitations in data availability, such as patients who developed BOS >5 years post-transplant or patients who died soon after BOS diagnosis (before their next yearly follow-up record), hampers the study’s generalizability. On the other hand, since these exclusions applied universally to all groups, they are unlikely to introduce major bias favoring or disfavoring any immunosuppressive regimen.

An important strength of the study is the use of the national SRTR dataset containing all U.S. lung transplant recipients, as this is one of the largest and most representative and comprehensive registries of BOS patients. However, this dataset unfortunately does not contain information on Restrictive Allograft Syndrome (RAS),^54^ which was relatively recently distinguished from BOS as a separate phenotype of CLAD that is much less common but more severe.^55^ Nevertheless, our subgroup analysis of “severely affected” BOS patients very likely contains significant numbers of patients whose condition overlapped with what would now be classified as RAS, as many patients in our study preceded the era when RAS had been well-distinguished from BOS. Finally, an additional strength is that our analyses adjusted for >20 important demographic, clinical, and transplant-related variables, including transplant center performance, to address the possibility that sirolimus tended to be used more frequently at centers that are inherently better-performing or worse-performing (regardless of which immunosuppressive drugs are used).

In summary, this study utilizing national U.S. lung transplant data suggests that for patients with BOS, sirolimus + tacrolimus may significantly improve survival over MMF + tacrolimus, while the combination of sirolimus + tacrolimus + MMF/azathioprine may further improve short-term survival, especially among patients who are severely affected. Considering the relatively small numbers of patients receiving sirolimus, and the retrospective, observational design and other associated limitations of this study, further studies are needed to confirm the favorable survival outcomes observed with sirolimus use in BOS patients. It would be especially useful for future studies to examine the following: 1) outcomes compared between sirolimus + tacrolimus + MMF/azathioprine and sirolimus + tacrolimus (the two best performing regimens in this study), 2) whether sirolimus + tacrolimus + MMF/azathioprine exhibits higher survival for the first 2–3 years after BOS onset while sirolimus + tacrolimus is more optimal for the long-term, as suggested by this study’s results, and 3) the optimal doses and/or trough levels of these drugs to balance efficacy and safety. Meanwhile, given the high mortality among BOS patients, use of the sirolimus and tacrolimus combination for maintenance immunosuppression (and adding MMF or azathioprine to this combination if possible, particularly in the short-term), should be carefully considered in each of these patients, to achieve the best chances of maximizing their survival.

## Disclosure

The authors of this manuscript have no conflicts of interest to disclose.

## Funding

This study was supported through: awards T32 AG000262 and P30 AG028747 from the National Institutes of Health, the Plylar family’s Love for Lungs Tennis Tournament, the Margaret Riehl Foundation, a Northwell Health Research Bridge Program Award, a Thomas and Marie Hales Foundation Award, and a gift by Ronald Antanasio (Recipient Aldo Iacono, MD).

## Declaration of competing interest

The authors declare that they have no known competing financial interests or personal relationships that could have appeared to influence the work reported in this paper.

## Data Availability

Data are available from the Scientific Registry of Transplant Recipients.

## References

[1] Gauthier Jason, Hachem Ramsey, Kreisel D. (2016). Update on chronic lung allograft dysfunction. Curr Transplant Rep.

[2] Verleden G.M., Vos R., Vanaudenaerde B. (2015). Current views on chronic rejection after lung transplantation. Transpl Int.

[3] Kulkarni H.S., Cherikh W.S., Chambers D.C. (2019). Bronchiolitis obliterans syndrome–free survival after lung transplantation: an International Society for Heart and Lung Transplantation Thoracic Transplant Registry analysis. J Hear Lung Transplant.

[4] Corris P.A., Ryan V.A., Small T. (2015). A randomised controlled trial of azithromycin therapy in bronchiolitis obliterans syndrome (BOS) post lung transplantation. Thorax.

[5] Reams B.D., Musselwhite L.W., Zaas D.W. (2007). Alemtuzumab in the treatment of refractory acute rejection and bronchiolitis obliterans syndrome after human lung transplantation. Am J Transplant.

[6] Hachem R.R., Patterson G.A., Trulock E.P. (2004). Efficacy of thymoglobulin® for the treatment of bos after lung transplantation and predictors of a therapeutic response. J Heart Lung Transplant.

[7] Iacono A., Wijesinha M., Rajagopal K. (2019). A randomised single-centre trial of inhaled liposomal cyclosporine for bronchiolitis obliterans syndrome post-lung transplantation. ERJ Open Res.

[8] Jain R., Hachem R.R., Morrell M.R. (2010). Azithromycin is associated with increased survival in lung transplant recipients with bronchiolitis obliterans syndrome. J Heart Lung Transplant.

[9] Kotecha S., Paul E., Ivulich S. (2021 Mar 16). Outcomes following ATG therapy for chronic lung allograft dysfunction. Transplant Direct.

[10] Padhye A.A., Guffey D., Leon-Pena A. (2025 Jul 4). Antithymocyte globulin therapy in chronic lung allograft dysfunction. Front Transplant.

[11] Bhorade S.M., Stern E. (2009). Immunosuppression for lung transplantation. Proc Am Thorac Soc.

[12] King-Biggs M.B., Dunitz J.M., Park S.J., Kay Savik S., Hertz M.I. (2003). Airway anastomotic dehiscence associated with use of sirolimus immediately after lung transplantation. Transplantation.

[13] Yanik E.L., Siddiqui K., Engels E.A. (2015 Sep). Sirolimus effects on cancer incidence after kidney transplantation: a meta-analysis. Cancer Med..

[14] Toso C., Merani S., Bigam D.L., Shapiro A.M., Kneteman N.M. (2010 Apr). Sirolimus-based immunosuppression is associated with increased survival after liver transplantation for hepatocellular carcinoma. Hepatology.

[15] Law B.K. (2005). Rapamycin: an anti-cancer immunosuppressant?. Crit Rev Oncol Hematol.

[16] Nair N., Gongora E., Mehra M.R. (2014). Long-term immunosuppression and malignancy in thoracic transplantation: where is the balance ?. J Hear Lung Transplant.

[17] Blagosklonny M.V. (2012). Rapalogs in cancer prevention Rapalogs in cancer prevention anti-aging or anticancer?. Cancer Biol Ther.

[18] Buchholz B.M., Ferguson J.W., Schnitzbauer A.A., International SiLVER study group (2020 May). Randomized sirolimus-based early calcineurin inhibitor reduction in liver transplantation: impact on renal function. Transplantation.

[19] Weir M.R., Mulgaonkar S., Chan L. (2011 Apr). Mycophenolate mofetil-based immunosuppression with sirolimus in renal transplantation: a randomized, controlled Spare-the-Nephron trial. Kidney Int.

[20] Gottlieb J., Neurohr C., Müller-Quernheim J. (2019 Jun 1). A randomized trial of everolimus-based quadruple therapy vs standard triple therapy early after lung transplantation. Am J Transplant.

[21] Demopoulos L., Polinsky M., Steele G. (2008 Jun). Reduced risk of cytomegalovirus infection in solid organ transplant recipients treated with sirolimus: a pooled analysis of clinical trials. Transplant Proc.

[22] Lamming D.W., Ye L., Sabatini D.M., Baur J.A. (2013). Review series Rapalogs and mTOR inhibitors as anti-aging therapeutics. J Clin Invest.

[23] Sehgal Suren N. (1998). "Rapamune®(RAPA, rapamycin, sirolimus): mechanism of action immunosuppressive effect results from blockade of signal transduction and inhibition of cell cycle progression.". Clin Biochem.

[24] Barten Markus J., Streit Frank, Boeger Martin (April 27, 2004). Synergistic effects of sirolimus with cyclosporine and tacrolimus: analysis of immunosuppression on lymphocyte proliferation and activation in rat whole blood. Transplantation.

[25] Vu Minh Diem, Qi Shijie, Xu Dasheng (December 27, 1997). Tacrolimus (FK506) and sirolimus (rapamycin) in combination are not antagonistic but produce extended graft survival in cardiac transplantation in the rat. Transplantation.

[26] Böhler T., Waiser J., Budde K. (1998). The in vivo effect of rapamycin derivative SDZ RAD on lymphocyte proliferation. Transplant Proc.

[27] Zhao Y., Gillen J.R., Meher A.K., Burns J.A., Kron I.L., Lau C.L. (2016 Feb). Rapamycin prevents bronchiolitis obliterans through increasing infiltration of regulatory B cells in a murine tracheal transplantation model. J Thorac Cardiovasc Surg.

[28] Song J., Du G., Chen W. (2020). The advantage of Sirolimus in amplifying regulatory B cells and regulatory T cells in liver transplant patients. Eur J Pharmacol.

[29] Bansal D., Yadav A.K., Kumar V., Minz M., Sakhuja V., Jha V. (2013). Deferred pre-emptive switch from calcineurin inhibitor to sirolimus leads to improvement in GFR and expansion of T Regulatory cell population: a randomized, controlled trial. PLoS ONE.

[30] Gillen J.R., Zhao Y., Harris D.A., LaPar D.J., Kron I.L., Lau C.L. (2013 Aug). Short-course rapamycin treatment preserves airway epithelium and protects against bronchiolitis obliterans. Ann Thorac Surg.

[31] Gillen J.R., Zhao Y., Harris D.A. (2013 May). Rapamycin blocks fibrocyte migration and attenuates bronchiolitis obliterans in a murine model. Ann Thorac Surg.

[32] Nair R.V., Huang X., Shorthouse R. (1997 Feb-Mar). Antiproliferative effect of rapamycin on growth factor-stimulated human adult lung fibroblasts in vitro may explain its superior efficacy for prevention and treatment of allograft obliterative airway disease in vivo. Transplant Proc.

[33] von Suesskind-Schwendi M., Brunner E., Hirt S.W. (2013 May). Suppression of bronchiolitis obliterans in allogeneic rat lung transplantation--effectiveness of everolimus. Exp Toxicol Pathol.

[34] Sacher V.Y., Fertel D., Srivastava K. (2014). Effects of prophylactic use of sirolimus on bronchiolitis obliterans syndrome development in lung transplant recipients. Ann Thorac Surg.

[35] Wijesinha M., Hirshon J.M., Terrin M. (2019). Survival associated with sirolimus plus tacrolimus maintenance without induction therapy compared with standard immunosuppression after lung transplant. JAMA Netw open.

[36] Strueber M., Warnecke G., Fuge J. (2016 Nov). Everolimus versus mycophenolate mofetil de novo after lung transplantation: a prospective, randomized, open-label trial. Am J Transplant.

[37] Bhorade S., Ahya V.N., Baz M.A. (2011). Comparison of sirolimus with azathioprine in a tacrolimus-based immunosuppressive regimen in lung transplantation. Am J Respir Crit Care Med.

[38] Snell G.I., Valentine V.G., Vitulo P. (2006 Jan). RAD B159 Study Group. Everolimus versus azathioprine in maintenance lung transplant recipients: an international, randomized, double-blind clinical trial. Am J Transplant.

[39] Kneidinger N., Valtin C., Hettich I. (2022 Sep 1). Five-year outcome of an early everolimus-based quadruple immunosuppression in lung transplant recipients: follow-up of the 4EVERLUNG study. Transplantation.

[40] Mariski M., Feist A., Yung G., Awdishu L. (2014). Sirolimus improves renal function and may prevent BOS progression after lung transplant. J Hear Lung Transplant.

[41] Wojarski J., Żegleń S., Ochman M., Karolak W. (2018). Early sirolimus-based immunosuppression is safe for lung transplantation patients: retrospective, single arm, exploratory study. Ann Transplant.

[42] Iturbe-Fernández D., de Pablo Gafas A., Mora Cuesta V.M. (2024). Everolimus treatment for chronic lung allograft dysfunction in lung transplantation. Life.

[43] Villanueva J., Boukhamseen A., Bhorade S.M. (2005). Successful use in lung transplantation of an immunosuppressive regimen aimed at reducing target blood levels of sirolimus and tacrolimus. J Hear Lung Transplant.

[44] Laporta Hernández R., Ussetti Gil P., García Gallo C., De Pablo Gafas A., Carreño Hernández M.C., Ferreiro Álvarez M.J. (2005). Rapamycin in lung transplantation. Transplant Proc.

[45] Cahill B.C., Somerville K.T., Crompton J.A. (2003). Early experience with sirolimus in lung transplant recipients with chronic allograft rejection. J Heart Lung Transplant.

[46] Groetzner J., Wittwer T., Kaczmarek I. (2006). Conversion to sirolimus and mycophenolate can attenuate the progression of bronchiolitis obliterans syndrome and improves renal function after lung transplantation. Transplantation.

[47] Vu M.D., Qi S., Xu D. (1998 Dec 27). Synergistic effects of mycophenolate mofetil and sirolimus in prevention of acute heart, pancreas, and kidney allograft rejection and in reversal of ongoing heart allograft rejection in the rat. Transplantation.

[48] Leppke S., Leighton T., Zaun D. (2013 Apr). Scientific registry of transplant recipients: collecting, analyzing, and reporting data on transplantation in the United States. Transplant Rev (Orlando)..

[49] Erdman Jay, Wolfram Josephine, Nimke David (June 2022). Lung transplant outcomes in adults in the United States: retrospective cohort study using real-world evidence from the SRTR. Transplantation.

[50] Sugihara M. (2010). Survival analysis using inverse probability of treatment weighted methods based on the generalized propensity score. Pharm Stat.

[51] Lachin J.M. (1999). Worst-rank score analysis with informatively missing observations in clinical trials. Control Clin Trials.

[52] Wang K., Zhang H., Li Y. (2004 Sep). Safety of mycophenolate mofetil versus azathioprine in renal transplantation: a systematic review. Transplant Proc.

[53] Snell G.I., Ennis S.L., Levvey B.J. (2024). Inhaled immunosuppressants after lung transplantation –real potential to enhance patient outcomes. Expert Rev Respir Med.

[54] Sato M., Waddell T.K., Wagnetz U. (2011 Jul). Restrictive allograft syndrome (RAS): a novel form of chronic lung allograft dysfunction. J Heart Lung Transplant.

[55] Verleden S.E., Hendriks J.M.H., Lauwers P., Yogeswaran S.K., Verplancke V., Kwakkel-Van-Erp J.M. (2023 Feb 1). Biomarkers for chronic lung allograft dysfunction: ready for prime time. Transplantation.

